# Assessment of Oral Hygiene Practices and Dental Health Conditions in School-Aged Children of 7–10 Years

**DOI:** 10.3390/children12101288

**Published:** 2025-09-24

**Authors:** Ana-Gabriela Seni, Liana Todor, Andreea Mihaela Kis, Mădălina-Gabriela Cincu, Ramona Amina Popovici, Anca Porumb, Iustin Olariu, Monica Tarcea

**Affiliations:** 1 Doctoral School, Faculty of Medicine, “George Emil Palade” University of Medicine, Pharmacy, Science and Technology of Târgu Mureș, 540139 Târgu Mureș, Romania; gabriela.seni@umfst.ro; 2 Departament of Dental Medicine, Faculty of Medicine and Pharmacy, University of Oradea, 10 Decembrie Sq., 410068 Oradea, Romania; liana.todor@gmail.com (L.T.); anca.porumb@yahoo.com (A.P.); 3 Research Center for Pharmaco-Toxicological Evaluations, Faculty of Pharmacy, “Victor Babes” University of Medicine and Pharmacy, Eftimie Murgu Sq., No. 2, 300041 Timisoara, Romania; kis.andreea@umft.ro; 4 Faculty of Medicine, “George Emil Palade” University of Medicine, Pharmacy, Science and Technology of Târgu Mures, 540142 Târgu Mureș, Romania; 5 Department of Management and Communication in Dental Medicine, Department I, Faculty of Dental Medicine, Victor Babes University of Medicine and Pharmacy of Timișoara, 300041 Timișoara, Romania; 6 Department of Dentistry, Faculty of Dentistry, “Vasile Goldiș” Western University of Arad, 310025 Arad, Romania; olariu.iustin@uvvg.ro; 7 Department of Community Nutrition, Faculty of Medicine, “George Emil Palade” University of Medicine, Pharmacy, Science and Technology of Târgu Mures, 540142 Târgu Mures, Romania; monica.tarcea@umfst.ro

**Keywords:** dental caries, periodontal disease, oral diseases, oral hygiene status, brushing

## Abstract

**Background/Objectives**: The ongoing prevalence of dental issues within the school-age population continues to present significant public health challenges. This study aims to thoroughly evaluate the oral health conditions of schoolchildren aged 7–10 years, with a particular focus on identifying the prevalence and severity of dental caries, as well as their relationship with dental hygiene and dietary habits. **Methods**: A cross-sectional approach was employed involving 700 children aged from 7 to 10 years, recruited from nine urban and rural educational institutions across two Romanian counties, namely Mureș and Bistrița-Năsăud. Data were collected regarding the sociodemographic characteristics of participants, including parental educational background and occupational status. Furthermore, information on dental hygiene routines was gathered, encompassing tooth brushing habits, flossing usage, frequency of brushing, along with dietary details such as daily meal count, snacking frequency, and sugar intake levels. An oral examination was performed by a trained specialist to assess the condition of the children’s teeth, documenting the number of decayed, missing, and filled teeth. The presence of plaque was evaluated using a plaque index, and the dental caries status was quantified using the Decayed, Missing, and Filled Teeth (DMFT) index. **Results**: The findings revealed that the average plaque score was marginally higher in boys (mean = 0.69 ± 0.36) compared to girls (mean = 0.65 ± 0.40). Additionally, children from urban environments demonstrated a mean plaque score of 0.61 ± 0.32, whereas their rural counterparts exhibited a higher score of 0.73 ± 0.38. In multivariable models, irregular brushing, higher daily sugar consumption, and ≥3 snacks/day were independently associated with both caries prevalence (DMFT > 0) and greater DMFT counts (*p* < 0.05)**. Conclusions**: The evidence denotes a concerning association between poor oral hygiene practices and higher rates of tooth decay among children, particularly among those with elevated sugar intake and frequent snacking behaviors. Dental public health professionals are encouraged to incorporate considerations of oral hygiene habits when developing future health promotion strategies aimed at improving the oral health status of children.

## 1. Introduction

Assessing oral hygiene and dental health among schoolchildren is extremely important, as this age group lays the foundation for lifelong oral health habits. The main factors influencing children’s oral hygiene are parental education and involvement. When parents have knowledge and understand the importance of dental health, the plaque index in their children decreases significantly. This relationship is largely attributed to improvements in parents’ behaviors regarding dental hygiene practices, which in turn are reflected in children’s practices [1,2]. In addition, the level of education attained by parents has been linked to the prevalence of dental caries in children; parents who are more educated usually show greater awareness of effective oral care, leading to lower cases of dental problems among their children [3]. Such findings suggest that parent-oriented educational programs could serve as an effective strategy for improving children’s oral health. Dental care anxiety is another key factor affecting children’s dental health. Children with poor oral health often have higher levels of dental anxiety [4]. Addressing dental care anxiety through educational measures and providing positive dental experiences could lead to better health outcomes and increased cooperation during dental visits [5]. Dental caries occurs because of demineralization of hard dental tissues, mainly attributed to the interaction of carbohydrates with bacterial biofilms in the oral cavity. The disease process begins when the bacteria that form plaque produce acids that dissolve minerals on the surface of the tooth, resulting in cavities. This process can be aggravated by various environmental and individual factors, including eating habits, oral hygiene practices, and inherent physiological characteristics [6,7]. Various demographic and socioeconomic factors play a key role in the prevalence and incidence of dental caries. Studies show that children and adolescents from lower socioeconomic backgrounds tend to have a higher prevalence of dental caries due to limited access to dental care, inadequate oral hygiene practices, and socioeconomic stressors that influence food choices [8,9]. Silva et al. highlighted a direct correlation between lower social status and increased prevalence of caries due to inadequate access to dental services, also emphasizing the role of maternal education in influencing children’s oral health behaviors [10]. These findings are reinforced by the finding of Medina-Solís et al. who showed that children from lower-income backgrounds made less use of dental services, aligning poor oral health with limited access to health care [11]. Parental education level and occupational status have been shown to correlate with caries prevalence, as parents with lower levels of education and income have children who are more likely to suffer from dental caries [12,13,14]. Dental hygiene practices and eating habits are other crucial factors influencing the incidence of tooth decay. Research underlines that poor oral hygiene, such as inadequate brushing techniques, contributes significantly to the prevalence of caries [15,16]. In addition, frequent consumption of sugary snacks has been identified as a risk factor for the development of dental caries, and dietary changes have been recommended to limit this exposure [15,17]. Therefore, the prevalence of dental caries in children remains a significant public health concern worldwide, with varying degrees of incidence in different populations. Research indicates that about 60–90% of children are affected by dental caries globally, making it one of the most common chronic diseases in pediatric populations [13,18]. Identifying periodontal problems and oral diseases in children is another crucial issue today, as it affects not only their oral health but also their overall quality of life. Periodontal diseases are a complex group of inflammatory conditions that affect the periodontium, including the supporting structures of the teeth, and are mainly caused by various forms of bacteria. Specifically, periodontitis is marked by the loss of periodontal attachment and is often initiated by the buildup of bacterial plaque on the teeth, leading to inflammation and the potential for irreversible tissue damage [19,20,21]. Gingivitis or periodontitis can be prevalent in pediatric populations and have the potential to cause serious consequences if left untreated [22,23]. Therefore, recognizing periodontal disease at an early stage can prevent progression into more severe forms that may occur in late adolescence or early adulthood, thus underlining the importance of routine oral health assessments [24,25,26]. Early diagnosis of periodontal problems allows the development of personalized treatment plans specific to each child’s unique oral health needs. Parents and teachers play an essential role in keeping children’s oral health. Understanding the relationship between a child’s oral health and overall well-being can improve proactive prevention and treatment measures. Therefore, prioritizing oral health during children’s formative years leads to improved immediate health outcomes, paving the way to a healthier future. In the context of the above, the present study aimed to assess the oral-dental status of 700 children aged 7 to 10 years from urban and rural areas of two counties of Romania, identifying the prevalence and severity of dental caries alongside their relationship with dental hygiene and dietary habits.

## 2. Materials and Methods

### 2.1. Ethical Consideration, Study Design, and Sample Size

The research was carried out with the authorization of the Scientific Research Ethics Committee affiliated with the “George Emil Palade” University of Medicine, Pharmacy, Science and Technology in Târgu Mureş (approval number 3147, dated 20 May 2024). The participant pool consisted of 700 children, ranging from 7 to 10 years old, representing both genders. Healthy children were included in the study based on their dentition. The criteria for exclusion were established concerning mental health and physical illnesses (for instance, intellectual disabilities, muscular paralysis, cerebral palsy, autism spectrum disorders, and central nervous system disorders resulting from perinatal hypoxia, as well as chronic illnesses and conditions characterized by diminished saliva production, such as xerostomia). Children diagnosed with these conditions were omitted from the study to mitigate any possible impact from related health issues. Before the initiation of the study, parents were briefed about the objectives and procedures involved, and informed consent was secured accordingly. The school authorities, in Mureş and Bistriţa-Năsăud counties, also granted permission to conduct the study. Specialists from the “George Emil Palade” University of Medicine, Pharmacy, Sciences and Technology from Târgu Mureş visited the nine schools to collect data over 1 year (May 2024–May 2025). To uphold the uniformity of the study population and to guarantee both the consistency and efficacy of the analysis, the sample was restricted to children aged 7 to 10 from the two counties mentioned in Romania.

### 2.2. Data Collection

The children’s parents answered questions to draw the participant’s demographic profile (name, age, gender, location, education, and professional status of the parents). Classroom teachers contributed to the distribution and assistance in completing a questionnaire regarding the behavioral and educational analysis of children. Furthermore, the class teachers were present to offer clarifications on the inquiries posed in the surveys. This assistance aimed to promote candid and comprehensive responses from parents, reflecting the familiarity and rapport they had with their children’s educators. Each completed questionnaire and associated child evaluation was systematically documented using a distinct identification code, thereby maintaining the confidentiality of the data collected. The questionnaire also included questions regarding toothbrush use, brushing frequency, dental flossing, total number of meals per day, number of snacks between meals, and sugar consumption.

### 2.3. Clinical Screening

Children underwent an oral examination by a qualified pedodontist to detect caries or other oral problems. To ensure consistency and reproducibility of clinical caries assessments, the examiner underwent a calibration process before data collection began. The calibration procedure included a two-day training session based on the World Health Organization (WHO) diagnostic criteria for dental caries [27]. During this session, both theoretical definitions and practical clinical exercises were performed. For reliability testing, a subsample of 30 children, representing the age groups between 7 and 10 years, was pre-examined. Dental caries was recorded separately for the permanent and primary dentitions. For permanent teeth, we used the DMFT index (Decayed, Missing due to caries, and Filled Teeth). For primary teeth, we used the dmft index (decayed, missing due to caries, and filled primary teeth). Indices were computed at the child level as sums across teeth and summarized as both prevalence (proportion with index > 0) and severity (mean, SD; median, IQR).

Dental plaque was assessed using the Silness–Löe Plaque Index (PI) on index teeth, scored 0–3 per tooth and averaged per child; higher scores indicate more plaque. For interpretability, we classified PI into 0 = excellent, 0.1–0.9 = good, 1.0–1.9 = fair, and 2.0–3.0 = poor.

### 2.4. Statistical Analysis

The results obtained were entered into a database for evaluation, and their expression was as mean ± standard deviation (SD). Statistical evaluation was carried out using SPSS software version 27 (IBM Company, Armonk, NY, USA), and a *p* value < 0.05 was considered statistically significant.

The DMFT and dmft by age (7, 8, 9, 10 years) and sex were summarized. The Significant Caries Index (SIC) was computed as the mean DMFT (or dmft) among the one-third of children with the highest scores; polarization was expressed as SIC divided by the overall mean. Caries prevalence (DMFT > 0; dmft > 0) was modeled with modified Poisson with robust errors (aPR), and severity (DMFT; dmft counts) with negative binomial regression after assessing overdispersion. Models adjusted for age, sex, urban/rural residence, PI, brushing frequency, flossing, sugar consumption/day, snack frequency/day, meals/day, and parental education. Significance was set at 0.05 (two-sided). PI was analyzed as a continuous variable (mean ± SD) and as categories (excellent/good/fair/poor). We compared PI across age groups (7–8, 8–9, 9–10 years), gender, and location (urban/rural), reporting *p*-values for group differences.

The prevalence (% with DMFT > 0; % with dmft > 0) and severity (means ± SD) overall and by age groups were summarized. Bivariate associations used χ^2^ tests for prevalence and ANOVA for means. We then estimated adjusted prevalence ratios (aPR) for DMFT > 0 using modified Poisson regression with robust errors, including age group, sex, and residence as covariates; exposures entered as categories (reference = healthiest pattern).

## 3. Results

Table 1 shows the demographic profile of the study participants. It can be seen that out of the total of 700 participating children, 356 were boys and 344 were girls, divided by age groups as follows: age group 7–8 years (107 boys vs. 117 girls), age group 8–9 years (97 boys vs. 87 girls), and age group 9–10 years (152 boys vs. 140 girls). 141 boys and 155 girls came from urban areas, and 215 boys and 189 girls from rural areas. In terms of fathers’ education, the fathers of 217 boys and 194 girls have finished a university; the fathers of 54 boys and 81 girls graduated from high school, and the fathers of 87 boys and 67 girls have finished only secondary school (8 classes). Regarding the mother’s education, the mothers of 270 boys and 261 girls presented a high level of education, the mothers of 48 boys and 54 girls had finished high school, and the mothers of 40 boys and 27 girls had finished only secondary school. Overall, 301 fathers of boys and 295 fathers of girls were employed, compared with 61 mothers of boys and 97 mothers of girls who were employed.

Following statistical analysis of the demographic profile of the participants, we obtained a significant difference (*p* < 0.05) when the participants were compared according to the mother’s education, as well as to the parents’ occupation. Therefore, it seems that parents’ education and the environment in which they work daily significantly affect the general condition, including the oral health, of their children.

Across the indexed ages (7–8, 8–9, and 9–10 years), caries experience in the primary dentition (dmft) decreased with age, whereas caries in the permanent dentition (DMFT) increased. Overall, the mean dmft was 2.0 ± 2.0, and 56% of children had dmft > 0, while the mean DMFT was 0.66 ± 1.00 with 37% DMFT > 0. Age-specific means were: dmft 2.6 ± 2.1 (7–8 years), 2.1 ± 2.0 (8–9 years), and 1.5 ± 1.8 (9–10 years); DMFT 0.45 ± 0.80, 0.62 ± 0.95, and 0.85 ± 1.10, respectively. Medians and interquartile ranges are shown in Table 2.

**Figure 1:**
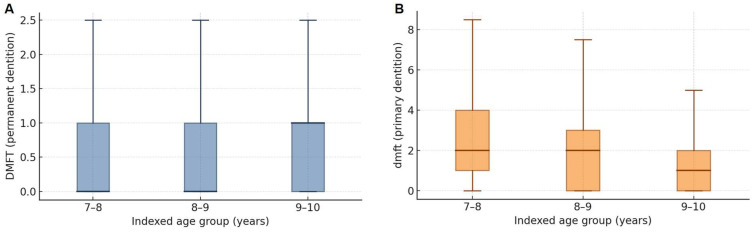
Distribution of caries experience by indexed age groups. (**A**) DMFT (permanent dentition) and (**B**) dmft (primary dentition) boxplots constructed from medians and IQRs reported in Table 2. Boxes show the interquartile range; horizontal lines indicate medians; whiskers follow the 1.5 × IQR rule (values truncated at 0).

To complement Table 2, Figure 1 displays boxplots of caries experience by indexed age. In the permanent dentition (DMFT), medians were 0 at 7–8 and 8–9 years and 1 at 9–10 years, with IQR [0–1] across ages. In the primary dentition (dmft), medians declined from 2 at 7–8 and 8–9 years to 1 at 9–10 years, with IQR [1–4], [0–3], and [0–2], respectively. These distributions illustrate the shift from primary- to permanent-tooth caries with age.

**Figure 2:**
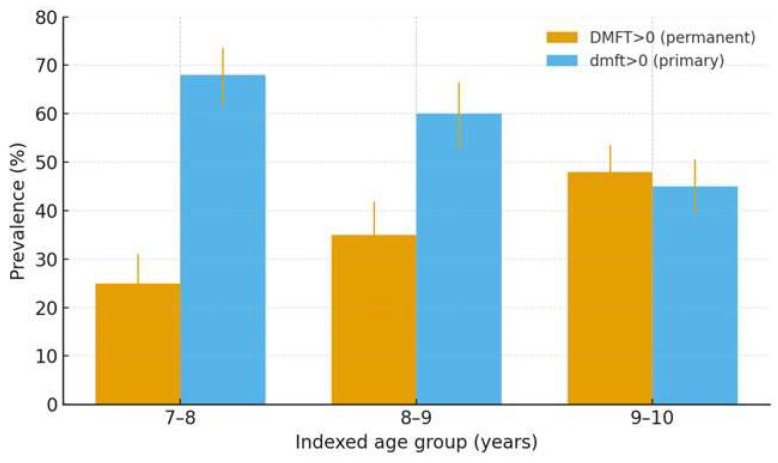
Prevalence of caries by indexed age groups with 95% CIs. Grouped bars show DMFT > 0 (permanent dentition) and dmft > 0 (primary dentition) at 7–8, 8–9, and 9–10 years. Error bars are 95% Wilson CIs computed from the age-specific sample sizes in Table 2.

Figure 2 displays the prevalence of caries in the permanent (DMFT > 0) and primary dentitions (dmft > 0) across indexed ages. Consistent with Table 2, DMFT > 0 increases from 25% at 7–8 years to 48% at 9–10 years, whereas dmft > 0 declines from 68% to 45% over the same range. Error bars indicate 95% confidence intervals (Wilson) based on the age-specific sample sizes.

Gender-stratified dmft in the primary dentition decreased with age in both sexes (Table 3). When sexes were combined (Table 2), the mean dmft declined from 2.6 at 7–8 years to 1.5 at 9–10 years, while the proportion with dmft > 0 fell from 68% to 45%, indicating a marked reduction in primary-tooth caries burden across indexed ages.

**Figure 3:**
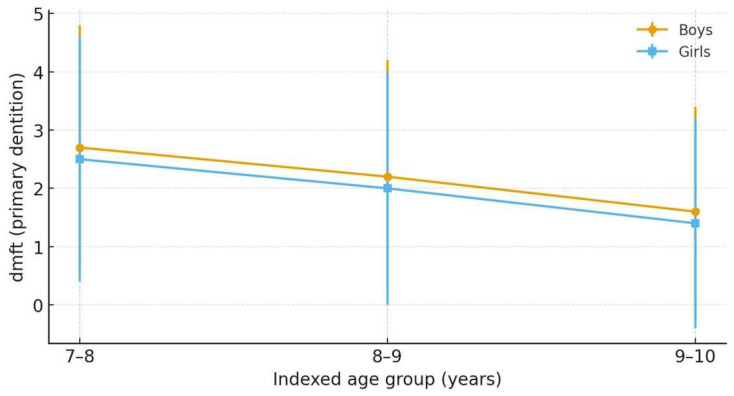
dmft in the primary dentition by indexed age and sex. Lines show mean dmft with ±SD error bars for boys and girls at 7–8, 8–9, and 9–10 years, using the summary statistics reported in Table 3.

Figure 3 depicts the primary-dentition dmft by indexed age and sex, shown as means with ± SD error bars (from Table 3). In both sexes, dmft decreases with age—from 2.7 → 1.6 in boys and 2.5 → 1.4 in girls (7–8 to 9–10 years). Across ages, boys exhibit slightly higher dmft than girls; differences are modest and consistent with the gender-stratified values in Table 3.

Age-specific SIC (mean ± SD) exceeded the corresponding mean in all groups, confirming polarization (Table 4); in the primary dentition SIC declined from 4.97 ± 2.2 at 7–8 years to 4.00 ± 2.0 at 9–10 years (polarization 1.92→2.67), whereas in the permanent dentition it increased from 1.35 ± 1.2 to 2.13 ± 1.5 (polarization 3.00→2.50), consistent with the transition from primary to mixed dentition.

**Figure 4:**
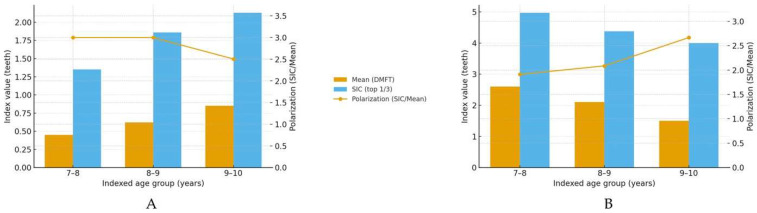
(**A**) DMFT (permanent dentition): Mean and age-specific SIC with polarization (SIC/Mean). Bars show the mean DMFT and the SIC (top one-third) for 7–8, 8–9, and 9–10 years; the overlaid line depicts polarization (right axis). (**B**) dmft (primary dentition): Mean and age-specific SIC with polarization (SIC/Mean). Bars show the mean dmft and the SIC (top one-third) by age; the overlaid line depicts polarization (right axis).

Figure 4 summarizes the Significant Caries Index (SIC) alongside the corresponding mean index within each age group and overlays the polarization (SIC/Mean). In the permanent dentition (DMFT), both the mean and SIC increase with age, while polarization declines modestly from ≈3.00 at 7–9 years to ≈2.50 at 9–10 years. In the primary dentition (dmft), the mean decreases with age, but SIC remains high, yielding higher polarization at 9–10 years (≈2.67), consistent with a concentrated burden among a minority of children as primary teeth exfoliate.

Table 5 presents the Silness–Löe Plaque Index (PI) by age, gender, and place of residence. The overall PI was 0.67 ± 0.41. By age, the mean plaque score increased progressively: 0.60 ± 0.30 at 7–8 years, 0.67 ± 0.43 at 8–9 years, and 0.73 ± 0.46 at 9–10 years, indicating a gradual rise in plaque accumulation across the indexed age groups. Consistent with this pattern, the distribution of PI categories (Table 5) shows a higher share of children with clinically relevant plaque (PI ≥ 1.0) at older ages. Regarding gender, boys exhibited a slightly higher mean PI than girls (0.69 ± 0.36 vs. 0.65 ± 0.40; *p* = 0.01). With respect to residence, children from rural areas recorded higher plaque levels than their urban counterparts (0.73 ± 0.38 vs. 0.61 ± 0.32; *p* = 0.02). These differences underscore modest but consistent gradients by age, sex, and environment.

**Figure 5:**
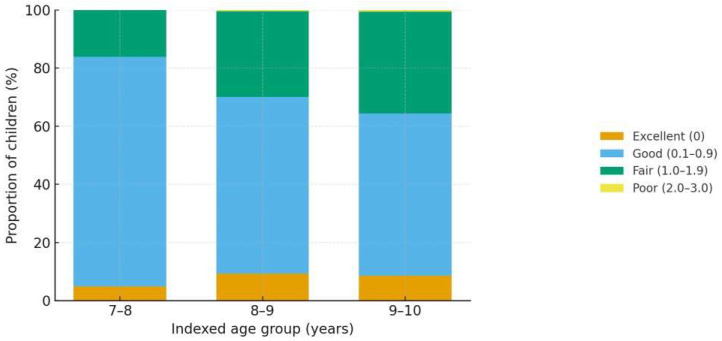
Plaque Index (PI) categories by indexed age groups (stacked bars). Bars show the proportions of Excellent (0), Good (0.1–0.9), Fair (1.0–1.9), and Poor (2.0–3.0) in 7–8, 8–9, and 9–10 years, based on the age-specific counts reported in Table 5. Minor rounding adjustments were made so that category totals match each age-group sample size exactly.

Figure 5 depicts the distribution of the Silness–Löe Plaque Index (PI) categories by indexed age groups. Consistent with Table 5, the proportion of children in the fair category (PI 1.0–1.9) increases with age, while the good category (0.1–0.9) correspondingly decreases; the poor category (2.0–3.0) remains rare across ages.

In Table 6, it is indicated that 563 children utilized toothpaste, resulting in an average plaque score of 0.75. In contrast, 137 children who employed dental floss achieved a plaque score of 0.88. Furthermore, among the cohort examined, 430 children reported brushing their teeth once per day, while 122 brushed twice daily, and 148 children brushed in an irregular manner. The corresponding mean plaque scores for these groups were recorded as 0.69, 0.60, and 0.75, respectively. Statistical analysis highlighted that these differences were significant, with a *p*-value of less than 0.05.

Therefore, using toothpaste, flossing, and brushing teeth at least once a day has a significant impact on dental health among schoolchildren and, therefore, can prevent the development of cavities or periodontal disease. The values regarding children’s eating habits, together with the values for the average score of decayed, missing, and filled teeth, are presented in Table 7.

The results show that the number of children with an eating frequency of 1–2 times per day was 61, 417 participants had an eating frequency of 2–3 times per day, and 222 participants declared that their eating frequency was above 4 times/day. The DMFT score according to the eating frequency was 0.61, 0.73, and 0.77, respectively. In addition, the results showed that 263 were low sugar usage children, 249 were medium usage, and 188 were high usage. The mean DMFT score according to the sugar usage per day was 0.55, 0.63, and 0.75, respectively. As concerns the administration of the snacks per day, the results showed that 245 children have a snack once/day; 280 children have snacks twice/day, and 175 children have snacks at least three times a day. The DMFT score according to the administration of the number of snacks/day was 0.56, 0.66, and 0.73, respectively. Also in this case, it seems that eating habits (especially daily sugar consumption (*p* = 0.01) and snacks between meals (*p* = 0.02)) significantly influence the occurrence of dental caries among schoolchildren.

**Figure 6:**
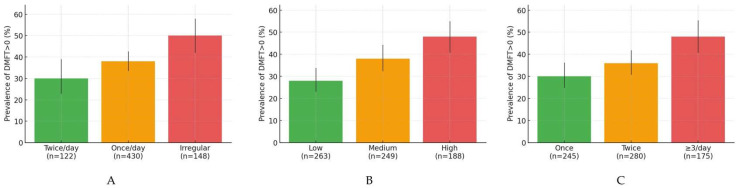
(**A**) Prevalence of DMFT > 0 by brushing frequency (with 95% CIs). Bars show the percentage of children with DMFT > 0 by twice/day, once/day, and irregular brushing. Error bars are 95% Wilson CIs using the sample sizes indicated under each bar. (**B**) Prevalence of DMFT > 0 by daily sugar intake (with 95% CIs). Bars show the prevalence by low, medium, and high daily sugar consumption; error bars denote 95% Wilson CIs. (**C**) Prevalence of DMFT > 0 by snacks/day (with 95% CIs). Bars show the prevalence by once, twice, and ≥3/day snacks; error bars denote 95% Wilson CIs.

Figure 6A–C depicts the bivariate relationships between caries prevalence in the permanent dentition (DMFT > 0) and key hygiene/diet behaviors. Prevalence increases stepwise with poorer brushing frequency (twice/day → once/day → irregular), with higher daily sugar intake (low → medium → high), and with more snacks per day (once → twice → ≥3/day). Error bars indicate 95% Wilson confidence intervals based on the category-specific sample sizes shown on the *x*-axis.

Table 8 shows the prevalence of caries in the permanent dentition (DMFT > 0) across hygiene and dietary categories. A clear graded pattern is evident: children who brush irregularly exhibit the highest prevalence, followed by those who brush once/day, with the lowest prevalence in those brushing twice/day. Similarly, prevalence rises from low to high daily sugar consumption and from 1 to ≥3 snacks per day. The gradient for the number of meals/day is modest by comparison. These bivariate findings mirror the severity trends reported in Table 7 (higher mean DMFT with poorer hygiene and less favorable diet) and support a dose–response relationship between cariogenic behaviors and caries presence.

Table 9 presents adjusted prevalence ratios (aPR) from modified Poisson models with robust errors, controlling for age group, sex, and residence. After adjustment, irregular brushing remains independently associated with higher caries prevalence compared with twice/day brushing, while once/day brushing shows a smaller, intermediate increase. High daily sugar intake is associated with higher prevalence compared with low intake, and consuming ≥3 snacks/day is likewise associated with elevated prevalence relative to one snack/day; the “twice/day” snack category shows a weaker or borderline association. These results indicate that both hygiene practices and cariogenic dietary behaviors contribute independently to caries risk, reinforcing the need for combined hygiene education and sugar/snacking reduction strategies.

**Figure 7:**
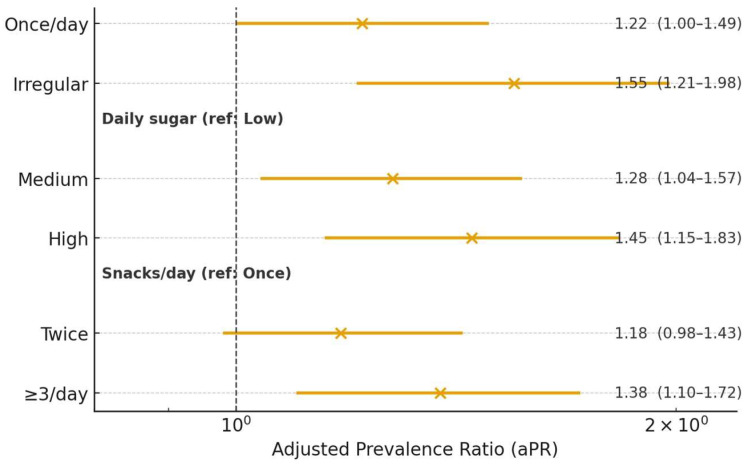
Adjusted prevalence ratios (aPR) for DMFT > 0 with 95% CIs. Forest plot of aPR (points) and 95% confidence intervals (horizontal lines) for brushing frequency (ref. twice/day), daily sugar intake (ref. low), and snacks/day (ref. once). Estimates correspond to Table 9; the dashed vertical line marks aPR = 1.0 (no association).

Figure 7 summarizes the adjusted associations between caries prevalence (DMFT > 0) and hygiene/diet behaviors. Estimates are adjusted prevalence ratios (aPR) from modified Poisson models with robust errors, controlling for age group, sex, and residence (Table 9). As shown, the risk is higher with irregular brushing (aPR 1.55, 95% CI 1.21–1.98), high daily sugar intake (1.45, 1.15–1.83), and ≥3 snacks/day (1.38, 1.10–1.72), while intermediate categories show smaller or borderline effects.

Multivariable analyses confirmed the patterns observed regarding the eating and oral hygiene habits. After adjustment for age, sex, residence, plaque index, brushing frequency, flossing, daily sugar consumption, snack frequency, number of meals/day, and parental education, irregular brushing, higher daily sugar consumption, and ≥3 snacks/day were associated with higher prevalence ratios of having caries (DMFT > 0) and with greater DMFT counts (all *p* < 0.05). Similar associations were observed for dmft.

## 4. Discussion

Analyzing the results of this cross-sectional study, we can state that, by assessing the current dental status of the pediatric population and correlating it with hygiene and dietary habits, this study makes important contributions in the field of pediatric oral health and suggests new directions for improving preventive interventions. The study included 700 children with a nearly even split between boys (356) and girls (344), categorized into age groups 7–8, 8–9, and 9–10 years, from both urban and rural areas; children from rural areas participated in the study in greater numbers. A significant difference (*p* < 0.05) was observed in the demographic profile based on the mother’s education and occupation, suggesting these factors influence children’s oral health. Caries experience in primary dentition (dmft index) decreased with age in both sexes from 7–8 to 9–10 years. Across ages, boys showed a slightly higher dmft index than girls. It has been stated that children in economically disadvantaged regions face considerable barriers to dental care due to a lack of awareness and lack of access to oral health education [28,29,30,31]. In economically underdeveloped regions, a lack of oral health awareness and education has been observed to further exacerbate the prevalence of dental caries among children [32,33,34,35,36]. Instead, holistic programs that integrate oral hygiene awareness into broader public health promotion can significantly reduce the burden of oral disease among these populations [37,38,39,40]. Such integration can include interventions in schools that actively involve both children and their parents, leading to improved oral hygiene behaviors. Our results are in agreement with the literature data. For example, in the study reported by Babu et al. [41], the authors determined the prevalence of dental caries and oral hygiene status in 1590 children (aged 7–12 years) of twenty schools in both rural and urban areas. The authors found a prevalence of dental caries of 65.6% among children (796 boys and 794 girls). Age groups 7–9 and 10–12 years of urban school children exhibited a higher prevalence of dental caries, and rural school children showed a poor oral hygiene status. Compared to boys and girls, the results of the authors showed that girls exhibit a higher prevalence of dental caries. Another research study found that socio-economic factors significantly correlated with higher caries rates in 6- and 12-year-olds, revealing both prevalence and severity reductions associated with increased access to preventive and restorative dental services [42,43]. Similarly, research in Kosovo emphasized that the prevalence of caries reflects regional disparities; schoolchildren’s dental caries was prevalent at alarming rates, further compounded by inadequate preventive measures and public health initiatives [44].

In our study, the results showed that the father’s education did not have as significant an impact on the child’s dental health as the mother’s education (*p* = 0.05 vs. *p* = 0.02 (Table 1). Numerous studies emphasize that a mother’s level of education and knowledge about dental health directly correlates with their children’s oral health behaviors and outcomes. Findings by Chen et al. suggest that mothers’ educational attainment confers a more substantial influence on family oral health practices than fathers’ [45]. This observation aligns with the argument that mothers are often the primary caregivers who manage day-to-day health decisions, including oral hygiene, eating habits, and attendance at dental visits for their children [2,46]. In addition, maternal involvement in health behaviors tends to predict positive dental health outcomes in children. Research indicates that mothers with higher awareness and knowledge about dental hygiene not only set better oral health standards for their children but also actively participate in health-promoting behaviors such as regular dental check-ups [47]. Therefore, while fathers play a critical role, particularly in terms of financial support for dental care, their involvement in daily health behaviors often does not match that of mothers in preventive oral health measures [48].

Furthermore, our results showed that in the primary dentition, SIC declined from 4.97 ± 2.2 at 7–8 years to 4.00 ± 2.0 at 9–10 years, while in the permanent dentition, it increased. The concept of caries polarization is crucial for understanding how dental caries manifests among distinct subsets of populations. Research has revealed a consistent pattern where a minority of children bear the majority of the caries burden, referred to as “the 80/20 rule,” in which 20% of children experience 80% of the decay. This phenomenon has been assessed through the Caries Assessment Spectrum and Treatment (CAST) index, elucidating variations in caries severity among children, influenced significantly by their environment and educational background [49,50]. This is pertinent when considering implications for oral health interventions aimed at decreasing disparities, indicating educational improvements concerning dental hygiene could have substantial public health impacts. In addition, inadequate oral hygiene practices contribute significantly to the higher rates of dental caries observed in populations with lower socioeconomic status. This can often be attributed to a lack of awareness or education about proper oral health practices among parents and caregivers [51]. This relationship is further complicated by eating habits influenced by socioeconomic stressors. Children from food-insecure families tend to consume diets high in carbohydrates and sugars, which favor the development of caries. Research indicates that these food choices are often driven by economic constraints, leading to poorer nutrition and subsequently poorer dental health outcomes. Studies have found that low socio-economic status is associated with dietary patterns that favor less nutritious choices, both due to financial limitations and lack of access to health-promoting foods [52,53].

Caries polarization is further emphasized through the use of the Simplified Oral Hygiene Index (SOPHI) and the Plaque Index. Studies demonstrate that despite knowledge about oral hygiene, such as tooth brushing frequency, there is often a gap in the practical application of this knowledge, with many children failing to brush effectively or regularly [54,55]. For example, while many children surveyed understood the necessity of regular brushing, many failed to adhere to this practice consistently, revealing a disconnect between knowledge and behavioral practices. This phenomenon is significant because correlations between poor oral hygiene habits and increased plaque indices have been established, leading to higher instances of gingivitis and caries [56]. Our results showed that the mean plaque score increased progressively with age, with boys exhibiting slightly higher mean PI than girls (*p* = 0.01), and rural children showing higher plaque levels than urban children (*p* = 0.02) (Table 5). Using toothpaste, flossing, and brushing teeth at least once a day has a significant impact on dental health among schoolchildren. In contrast with our study, Mahesh et al. [57] reported that there was no statistically significant difference in the DMFT score of boys and girls in the age groups participating in their study (5-year-olds vs. 12-year-olds).

Additionally, dietary habits play an instrumental role in the prevalence and severity of dental caries in children aged 7–10 years. Several studies have highlighted that increased consumption of sugar-rich snacks and beverages correlates strongly with the incidence of caries among young children. Some studies outlined that frequent intake of sugar-rich diets and fizzy drinks is a contributing factor leading to a higher incidence of dental issues during formative years [42,54]. This trend reiterates the importance of dietary education alongside oral hygiene education in combating childhood caries. In our study, we found that regarding eating habits, daily sugar consumption (*p* = 0.01) and snacks between meals (*p* = 0.02) significantly influence the occurrence of dental caries among schoolchildren (Table 7). These associations persisted after multivariable adjustment for demographic, hygiene, and dietary factors, indicating that irregular brushing, higher sugar intake, and frequent snacking are independent correlates of caries burden in this cohort. We found that children who brush irregularly exhibit the highest caries prevalence, followed by those who brush once/day. Caries prevalence rises with higher daily sugar consumption and more snacks per day. Irregular brushing, higher daily sugar consumption, and ≥3 snacks/day were associated with higher prevalence ratios of having caries (DMFT > 0) and with greater DMFT counts (all *p* < 0.05). Once again, our data correlates with other reports. For example, previous studies showed that increased frequency of snacks and sugar intake was associated with higher DMFT values, while irregular brushing was linked to more caries, in line with our results [58]. Other recent studies further emphasize the role of diet and microbiome [59], as well as broader systemic processes such as neuroinflammation, in shaping oral and general health [60].

Interventional studies have demonstrated that community-based oral health promotion projects can significantly enhance oral hygiene practices among schoolchildren. Programs designed to engage children with interactive and practical oral health education have successfully improved their brushing frequency and overall dental health awareness. In one longitudinal study conducted in Finland, a community intervention led to substantial improvements in children’s oral health knowledge and practices [61]. The data suggest that such comprehensive programs could serve as effective models for enhancing oral health prevention strategies across diverse educational settings. Moreover, the involvement of teachers and parents in reinforcing oral hygiene practices is crucial. Several studies indicate that parental influence is a key determinant in children’s dental health behavior. Children whose parents exhibited good oral hygiene habits were more likely to adopt similar practices, highlighting the importance of socio-cultural factors in dental health promotion [55,62]. This underscores the necessity of developing educational initiatives that resonate well within various family and community contexts.

A multi-factorial approach emphasizing parent education, child-centered interactive learning, and psychological support for dental anxiety is needed to improve the dental status and oral hygiene practices of school children aged 7–10 years. Efficient implementation of these strategies can reduce the prevalence of dental caries and improve the overall dental health of this vulnerable population. Educational interventions designed specifically for children have proven effective in improving their oral health knowledge and subsequent oral health behaviors. Studies have reported that the use of engaging educational materials, such as animated videos and storytelling, can positively influence children’s attitudes towards dental hygiene and their willingness to participate in regular brushing habits [63,64,65,66,67,68]. Research indicates that children who engage in interactive learning experiences about dental care are more likely to maintain healthier dental practices outside the classroom environment. In the present study, there were 700 schoolchildren, with ages ranging from 7 to 10 years, of both genders.

In line with the results obtained in the present study, we believe that educational interventions aimed at parents and children will improve oral hygiene awareness and behaviors, thereby potentially reducing the incidence of periodontal disease in children. In addition, initiatives that emphasize the importance of oral health during early childhood can be effective, as early habits often influence long-term oral health outcomes [69,70]. Recent literature also highlights the importance of psychosocial factors, effective communication, and the use of modern methods such as virtual reality in improving both oral and general health [71,72,73,74,75,76,77]. In addition, recent studies highlight the influence of contextual and behavioral factors on children’s oral health. The COVID-19 pandemic was associated with a deterioration of pediatric oral health, and the oral hygiene profile of Romanian schoolchildren indicates the need for preventive strategies adapted to local environments [78,79]. Socio-behavioral determinants remain strongly linked to caries status, while structured programs can improve oral hygiene behaviors in school-aged children [80,81]. Other findings emphasize the impact of environmental and lifestyle conditions, the effectiveness of interactive educational methods, the decisive role of parental involvement, and the value of preventive programs targeting mothers, even when delivered by non-dental professionals [82,83,84,85].

Taken together, these findings reinforce the importance of comprehensive, multi-level strategies that combine early education, psychosocial support, and contextual interventions to improve oral health in school-aged children.

## 5. Limitations of the Study

This study has several limitations that should be considered when interpreting the findings. First, the cross-sectional design precludes causal inference; the observed associations between hygiene/dietary behaviors and caries could be bidirectional or confounded by unmeasured factors.

Second, external validity is limited because participants were drawn from nine schools in two counties (Mureș and Bistrița-Năsăud, Romania) rather than from a nationally representative sample; selection and participation bias at the school or family level cannot be ruled out.

Third, behavioral exposures were parent/child-reported (tooth-brushing, flossing, meals/snacks, and sugar intake) and therefore are subject to recall and social-desirability bias; moreover, we did not apply a validated oral-health knowledge scale, so “knowledge and practice” were operationalized through reported behaviors rather than a separate knowledge score.

Fourth, caries was assessed clinically in school settings without adjunctive radiography, which likely led to under-detection of approximal/early lesions (a conservative bias in prevalence and severity estimates).

Fifth, residual confounding is possible: while models adjusted for key covariates (age, sex, residence, PI, brushing/flossing, sugar, snacks, meals, parental education), data on other determinants (e.g., fluoride exposure/sealants, beverage patterns, dental service use) were not collected, and clustering by school was not explicitly modeled, which could narrow confidence intervals.

Finally, data collection spanned one year (May 2024–May 2025); unmeasured period or seasonal effects in diet and routines may have influenced results.

## 6. Conclusions

This study set out to assess the oral–dental status of schoolchildren aged 7–10 years and to identify the prevalence and severity of dental caries alongside their relationship with dental hygiene and dietary habits. In line with this objective, our data show that caries burden is substantial in this age band and follows the expected age pattern: DMFT (permanent) tends to increase with age, while dmft (primary) decreases as primary teeth exfoliate. Dental plaque levels (PI) were modest on average but rose across age groups and were higher in rural than urban settings, with small gender differences, reflecting actionable gaps in daily oral hygiene. Caries concentration (SIC) exceeded the age-specific mean in all groups, indicating polarization of disease within each class and highlighting the need for targeted outreach to high-risk children. Finally, unfavorable hygiene and diet behaviors—irregular brushing, higher daily sugar intake, and frequent snacking (≥3/day)—were consistently associated with greater caries prevalence and higher DMFT counts, even after adjustment for key covariates. Together, these findings meet the study objective and support a two-tier strategy: (1) universal school-based measures (supervised toothbrushing with fluoride, sugar-reduction messaging) and (2) targeted interventions for the high-risk tertile identified by age-specific indices (e.g., sealants for first permanent molars and intensified preventive recall).

Our results support a two-tier approach that combines universal school-based measures with targeted actions for the high-risk tertile identified by SIC:

- School oral-health education and supervised toothbrushing: brief, weekly classroom modules (5–10 min.) on brushing and sugar consumption, plus daily supervised toothbrushing with fluoride toothpaste (1000–1450 ppm) in grades 1–4. Provide take-home kits at the beginning of each term.
- Targeted sealants and fluoride varnish: prioritize first permanent molars soon after eruption (≈7–8 years) for pit-and-fissure sealants; apply fluoride varnish 2–4×/year to children in the top SIC tertile or with PI ≥ 1.0. Use school dental teams/mobile units for rural schools.
- Risk-based recall and minimally invasive care: schedule 6-month recalls for high-risk children (SIC tertile, irregular brushing, high sugar/≥3 snacks/day) and use sealants where appropriate.
- Dietary environment in schools: adopt “water-only” and “sugar-smart” policies (limit sugary drinks/snacks at school/vending; encourage fruit/dairy snacks; align canteen offerings with low-sugar standards).
- Family engagement: brief parental counseling at school events or via leaflets/messages on twice-daily brushing and reducing daily sugar/snacks.
- Monitoring and equity: implement a simple risk screen (age band + DMFT/dmft > 0 + PI) at the class level, track SIC/polarization annually, and prioritize rural schools, where PI was higher.

These steps translate our age-specific DMFT/dmft patterns, higher PI with age and in rural settings, and SIC polarization into implementable actions that can reduce both overall caries burden and its concentration in high-risk groups.

## Figures and Tables

**Table 1:** Demographic profile.

Parameter	Boys (n = 356)	Girls (n = 344)	*p*
Age group (years)			
7–8	107	117	0.81
8–9	97	87	0.84
9–10	152	140	0.05
Location			
Urban	141	155	0.05
Rural	215	189	
Father education			
University	217	194	0.05
High school	54	81	
Secondary school	87	67	
Mother education			
University	270	261	0.02
High school	48	54	
Secondary school	40	27	
Father occupation			
Employed	301	295	0.15
Unemployed	57	47	
Mother occupation			
Employed	61	97	0.01
Unemployed	297	245	

**Table 2:** Caries experience in permanent (DMFT) and primary (dmft) dentitions by indexed age groups (7–10 years).

Age (Years)	n	DMFT Mean ± SD	DMFT Median [IQR]	DMFT > 0%	dmft Mean ± SD	dmft Median [IQR]	dmft > 0%
7–8	224	0.45 ± 0.80	0 [0–1]	25%	2.6 ± 2.1	2 [1–4]	68%
8–9	184	0.62 ± 0.95	0 [0–1]	35%	2.1 ± 2.0	2 [0–3]	60%
9–10	292	0.85 ± 1.10	1 [0–1]	48%	1.5 ± 1.8	1 [0–2]	45%
Overall	700	0.66 ± 1.00	0 [0–1]	37%	2.0 ± 2.0	2 [0–3]	56%

DMFT = Decayed, Missing (due to caries), Filled permanent teeth; dmft = decayed, missing, filled primary teeth. Prevalence = proportion with index > 0.

**Table 3:** dmft in primary dentition by indexed age groups and gender.

Age (Years)	Boys, n	Boys dmft Mean ± SD	Boys dmft Median [IQR]	Boys dmft > 0%	Girls, n	Girls dmft Mean ± SD	Girls dmft Median [IQR]	Girls dmft > 0%
7–8	107	2.7 ± 2.1	2 [1–4]	70%	117	2.5 ± 2.1	2 [1–4]	66%
8–9	97	2.2 ± 2.0	2 [0–3]	61%	87	2.0 ± 2.0	2 [0–3]	59%
9–10	152	1.6 ± 1.8	1 [0–2]	46%	140	1.4 ± 1.8	1 [0–2]	44%
Overall	356	2.1 ± 2.0	2 [0–3]	57%	344	1.9 ± 2.0	2 [0–3]	55%

**Table 4:** SIC by dentition and indexed age groups.

Dentition	Age Group	Mean Index	SIC (Mean ± SD)	SIC Polarization (SIC/Mean)
DMFT (permanent)	7–8 years	0.45	1.35 ± 1.2	3.00
	8–9 years	0.62	1.86 ± 1.4	3.00
	9–10 years	0.85	2.13 ± 1.5	2.50
	overall	0.66	2.00 ± 1.4	3.03
dmft (primary)	7–8 years	2.60	4.97 ± 2.2	1.92
	8–9 years	2.10	4.38 ± 2.1	2.08
	9–10 years	1.50	4.00 ± 2.0	2.67
	overall	2.00	4.46 ± 2.1	2.23

**Table 5:** Silness–Löe Plaque Index (PI) in children, by age group, gender, and location.

Parameter	n	PI Mean ± SD	*p*	Excellent (0) n (%)	Good (0.1–0.9) n (%)	Fair (1.0–1.9) n (%)	Poor (2.0–3.0) n (%)
Age (years)							
7–8 years	224	0.60 ± 0.30	0.05	11 (4.8%)	178 (79.4%)	36 (15.9%)	0 (0.0%)
8–9 years	184	0.67 ± 0.43		17 (9.2%)	112 (61.1%)	54 (29.4%)	0 (0.2%)
9–10 years	292	0.73 ± 0.46		25 (8.5%)	163 (55.9%)	102 (35.0%)	2 (0.5%)
overall	700	0.67 ± 0.41		53 (7.6%)	453 (64.7%)	192 (27.4%)	2 (0.3%)
Gender							
Boys	356	0.69 ± 0.36	0.01	18 (5.1%)	239 (67.1%)	99 (27.8%)	0 (0.0%)
Girls	344	0.65 ± 0.40		29 (8.4%)	224 (65.1%)	91 (26.5%)	0 (0.0%)
Location							
Urban	296	0.61 ± 0.32	0.02	16 (5.4%)	226 (76.4%)	54 (18.2%)	0 (0.0%)
Rural	404	0.73 ± 0.38		20 (5.0%)	252 (62.4%)	132 (32.7%)	0 (0.0%)

**Table 6:** The mean plaque score depending on oral hygiene habits.

Variable	N	Plaque Score	*p*
Oral hygiene habits			
Toothpaste	563	0.75	0.02
Flossing	137	0.88	0.04
Brushing frequency			
Once	430	0.69	0.01
Twice	122	0.6	
Irregular	148	0.75	

**Table 7:** The mean value of dietary habits, alongside the mean values of decayed, missing, and filled teeth scores.

Dietary Habits	N	DMFT Score	*p*
Eating frequency/day			
1–2	61	0.61	0.02
3–4	417	0.73	
>4	222	0.77	
Sugar consumption level/day			
Low	263	0.55	0.01
Medium	249	0.63	
High	188	0.75	
Snacks between meals/day			
Once	245	0.56	0.02
Twice	280	0.66	
≥3	175	0.73	

**Table 8:** Caries prevalence (DMFT > 0) by dental hygiene and dietary habits (bivariate).

Exposure	Categories (n)	DMFT > 0%
Brushing frequency	Twice/day (122)	30%
	Once/day (430)	38%
	Irregular (148)	50%
Daily sugar consumption	Low (263)	28%
	Medium (249)	38%
	High (188)	48%
Snacks between meals/day	Once (245)	30%
	Twice (280)	36%
	≥3 (175)	48%
Meals/day	1–2 (61)	31%
	3–4 (417)	36%
	>4 (222)	41%

**Table 9:** Adjusted associations with caries prevalence (DMFT > 0): modified Poisson models (age-, sex-, residence-adjusted).

Exposure (Reference)	Category	aPR	95% CI	*p*
Brushing (twice/day)	Once/day	1.22	1.00–1.49	0.04
	Irregular	1.55	1.21–1.98	0.001
Daily sugar (low)	Medium	1.28	1.04–1.57	0.02
	High	1.45	1.15–1.83	0.002
Snacks/day (once)	Twice	1.18	0.98–1.43	0.08
	≥3	1.38	1.10–1.72	0.005

## Data Availability

The data used and/or analyzed during the current study are available from the corresponding author upon reasonable request. Unfortunately, these data are not publicly available because of privacy or ethical restrictions.

## Abbreviations

The following abbreviations are used in this manuscript:

aPR	Adjusted prevalence ratios
CAST	Caries Assessment Spectrum and Treatment index
CI	Confidence interval
DMFT	Decayed, Missing, and Filled Teeth for permanent dentition
dmft	decayed, missing, and filled teeth for primary dentition
IQR	Interquartile
N	Number of study participants
PI	Silness–Löe Plaque Index
SD	Standard Deviation
SIC	Significant Caries Index
SOPHI	Simplified Oral Hygiene Index
WHO	World Health Organization

## References

[1] Salikun S., Supriyana S., Suwarsono S., Kusmana A., Setiadi Y. (2021). Integrated dental health monitoring using smart tooth brush and application. J. Health Med. Sci..

[2] Setiawati F., Darwita R., Fadillah F., Adiatman M., Soekanto S., Sjarkawi G. (2020). Effectiveness of dental health education intervention using the 16-surface tooth brushing program among 7-9-year-old schoolchildren in Indonesia. Pesqui. Bras. Em Odontopediatria E Clínica Integr..

[3] Liu Y., Zhu J., Zhang H., Jiang Y., Wang H., Yu J., Da D., Chen Q., Su H., Wu Z. (2024). Dental caries status and related factors among 5-year-old children in Shanghai. BMC Oral Health.

[4] Soares F.C., Lima R.A., Salvador D.M., de Barros M.V.G., Dahllöf G., Colares V. (2020). Reciprocal longitudinal relationship between dental fear and oral health in schoolchildren. Int. J. Paediatr. Dent..

[5] Daneshvar S., Azizi S. (2021). The relationship between dental fear and cooperation of children during dental treatments with their parents’ general health. Dentistry 3000.

[6] He J., Liang H., Kang J., Yuan C. (2022). Subgroup detection-based dental caries status and inequalities trend exploration: A nationwide, 10-year-repeated cross-sectional study. Front. Public Health.

[7] Meng Y., Wu T., Billings R., Kopycka-Kedzierawski D.T., Xiao J. (2019). Human genes influence the interaction between Streptococcus mutans and host caries susceptibility: A genome-wide association study in children with primary dentition. Int. J. Oral Sci..

[8] Ju X., Do L., Ha D., Jamieson L. (2019). Association of Modifiable Risk Factors with Dental Caries Among Indigenous and Nonindigenous Children in Australia. JAMA Netw. Open.

[9] Chen R., Schneuer F.J., Irving M.J., Chow C.K., Kumar H., Tsai C., Sohn W., Spallek H., Bell J., Nassar N. (2022). Socio-demographic and familial factors associated with hospital admissions and repeat admission for dental caries in early childhood: A population-based study. Community Dent. Oral Epidemiol..

[10] da Silva A.N., Mendonça M.H., Vettore M.V. (2011). The association between low-socioeconomic status mothers’ Sense of Coherence and their child’s utilization of dental care. Community Dent. Oral Epidemiol..

[11] Medina-Solis C.E., Maupomé G., del Socorro H.M., Pérez-Núñez R., Avila-Burgos L., Lamadrid-Figueroa H. (2008). Dental health services utilization and associated factors in children 6 to 12 years old in a low-income country. J. Public Health Dent..

[12] Kato H., Tanaka K., Shimizu K., Nagata C., Furukawa S., Arakawa M., Miyake Y. (2017). Parental occupations, educational levels, and income and prevalence of dental caries in 3-year-old Japanese children. Environ. Health Prev. Med..

[13] Nota A., Caruso S., Cantile T., Gatto R., Ingenito A., Tecco S., Ferrazzano G.F. (2019). Socioeconomic Factors and Oral Health-Related Behaviours Associated with Dental Caries in Preschool Children from Central Italy (Province of Ascoli Piceno). Biomed. Res. Int..

[14] Julihn A., Soares F.C., Hjern A., Dahllöf G. (2018). Socioeconomic Determinants, Maternal Health, and Caries in Young Children. JDR Clin. Trans. Res..

[15] Jain M., Namdev R., Bodh M., Dutta S., Singhal P., Kumar A. (2015). Social and Behavioral Determinants for Early Childhood Caries among Preschool Children in India. J. Dent. Res. Dent. Clin. Dent. Prospects.

[16] Ibrahim R.E.H.M., Helaly M.O., Ahmed E.M.A. (2021). Assessment of Brushing Techniques in School Children and Its Association with Dental Caries, Omdurman, 2019. Int. J. Dent..

[17] Emmanuelli B., Knorst J.K., Menegazzo G.R., Mendes F.M., Ardenghi T.M. (2021). The Impact of Early Childhood Factors on Dental Caries Incidence in First Permanent Molars: A 7-Year Follow-Up Study. Caries Res..

[18] Kumar S., Tadakamadla J., Kroon J., Johnson N.W. (2016). Impact of parent-related factors on dental caries in the permanent dentition of 6-12-year-old children: A systematic review. J. Dent..

[19] Li Y., Wu X., Liu M., Deng K., Tullini A., Zhang X., Shi J., Lai H., Tonetti M.S. (2024). Enhanced control of periodontitis by an artificial intelligence-enabled multimodal-sensing toothbrush and targeted mHealth micromessages: A randomized trial. J. Clin. Periodontol..

[20] Sălcudean A., Nan A.G., Bodo C.R., Cosma M.C., Strete E.G., Lica M.M. (2023). Association between Childhood Onset Inflammatory Bowel Disease and Psychiatric Comorbidities in Adulthood. Diagnostics.

[21] Wang X., Zou Y., Zhang J. (2024). Identification of biomarkers related to prognosis and diagnosis of periodontitis by bioinformatics based on public database. Oral Dis..

[22] Rai J., Pandrakula S., Bhadouria P., Shrivastava J., Srinivasan K., Payak A., Mehta D. (2023). Knowledge and attitude of primary school teachers for periodontal disease. J. Adv. Zool..

[23] Sağlam G., Dağ A. (2023). Gingival and periodontal diseases in children. J. Dent. Sci. Educ..

[24] Fardal Ø., Skau I., Grytten J. (2020). Familial tendency as a determinant of tooth loss during long-term periodontal therapy. J. Clin. Periodontol..

[25] Tilinca M.C., Antal C., Salcudean A., Abalasei B.L., Farcas R.M., Grosan A. (2023). New directions in pharmacological treatment with SGLT-2 inhibitor molecules in the light of current guidelines for diabetes mellitus, heart failure and kidney disease. Farmacia.

[26] Tilinca M.C., Merlan I., Sălcudean A., Tilea I., Nemes-Nagy E. (2021). Oxidative Stress and Cytokines’ Involvement in the Occurence and Progression of Diabetic Complications in the Covid-19 Pandemic Context. Farmacia.

[27] World Health Organization (1997). Oral Health Surveys: Basic Methods.

[28] Li S., Fan L., Zhou S. (2024). Analysis of the incidence and influencing factors of dental caries and periodontitis in children aged 5–12 in Jinhua, Zhejiang province. J. Clin. Pediatr. Dent..

[29] Răchită A., Strete G.E., Suciu L.M., Ghiga D.V., Sălcudean A., Mărginean C. (2022). Psychological Stress Perceived by Pregnant Women in the Last Trimester of Pregnancy. Int. J. Environ. Res. Public Health.

[30] Tilinca M.C., Antal C., Balint A., Sălcudean A., Varga A. (2023). The Newest Therapeutically Approach Of “Diabesity” Using Glp-1 Ra Molecules: Impact of the Oral Formulation. Farmacia.

[31] Bodo C.R., Salcudean A., Nirestean A., Lukacs E., Lica M.M., Muntean D.L., Anculia R.C., Popovici R.A., Neda Stepan O., Enătescu V.R. (2024). Association between Chronic Misophonia-Induced Stress and Gastrointestinal Pathology in Children—A Hypothesis. Children.

[32] Dhar V., Jain A., van Dyke T.E., Kohli A. (2007). Prevalence of dental caries and treatment needs in the school-going children of rural areas in Udaipur district. J. Indian. Soc. Pedod. Prev. Dent..

[33] Kalinovic R., Pascariu A., Vlad G., Nitusca D., Sălcudean A., Sirbu I.O., Marian C., Enatescu V.R. (2024). Involvement of the Expression of G Protein-Coupled Receptors in Schizophrenia. Pharmaceuticals.

[34] Salcudean A., Lica M.M. (2024). The Role of Systemic Family Psychotherapy in Glycemic Control for Children with Type 1 Diabetes. Children.

[35] Salcudean A., Strete G., Enatescu V.R., Tilinca M.C., Sasu A.B., Ceana D.E., Kiss M., Dumache R., Ősz B.E., Teodorescu D.R. (2024). Pilot Study to evaluate adherence to treatment using the Mars Scale on a sample of Romanian patients with cardiovascular diseases. Farmacia.

[36] Salcudean A., Osz B.E., Nan A.G., Sasu A.B., Cozma M.M., Tilinca M.C., Bocsak-Pop A.R., Strete E.G. (2023). The efficacy of antidepresive treatment on COVID-19 pandemic-related depressive symptoms-A prospective, single-center, questionnaire-based study. Farmacia.

[37] Duijster D., Monse B., Dimaisip-Nabuab J., Djuharnoko P., Heinrich-Weltzien R., Hobdell M., Kromeyer-Hauschild K., Kunthearith Y., Mijares-Majini M.C., Siegmund N. (2017). ‘Fit for school’—A school-based water, sanitation and hygiene programme to improve child health: Results from a longitudinal study in Cambodia, Indonesia and Lao PDR. BMC Public Health.

[38] Lica M.M., Papai A., Salcudean A., Crainic M., Covaciu C.G., Mihai A. (2021). Assessment of Psychopathology in Adolescents with Insulin-Dependent Diabetes (IDD) and the Impact on Treatment Management. Child.

[39] Răchită A.I.C., Strete G.E., Sălcudean A., Ghiga D.V., Huțanu A., Muntean L.M., Suciu L.M., Mărginean C. (2023). The Relationship between Psychological Sufferings, Value of Maternal Cortisol during Third Trimester of Pregnancy and Breastfeeding Initiation. Medicina.

[40] Herlo L.F., Salcudean A., Sirli R., Iurciuc S., Herlo A., Nelson-Twakor A., Alexandrescu L., Dumache R. (2024). Gut Microbiota Signatures in Colorectal Cancer as a Potential Diagnostic Biomarker in the Future: A Systematic Review. Int. J. Mol. Sci..

[41] Babu M.M., Nirmala S., Sivakumar N. (2011). Oral hygiene status of 7–12 year old school children in rural and Urban population of Nellore district. J. Indian. Assoc. Public Health Dent..

[42] Jindal L., Dua P., Mangla R., Gupta K., Vyas D., Gupta P. (2020). Dental Caries in Relation to Socioeconomic Factors of 6 and 12-year-old Schoolchildren of Paonta Sahib, Himachal Pradesh, India: An Epidemiological Study. Int. J. Clin. Pediatr. Dent..

[43] Shailee F., Girish M.S., Kapil R.S., Nidhi P. (2013). Oral health status and treatment needs among 12- and 15-year-old government and private school children in Shimla city, Himachal Pradesh, India. J. Int. Soc. Prev. Community Dent..

[44] Ferizi L., Dragidella F., Staka G., Bimbashi V., Mrasori S. (2017). Oral Health Status Related to Social Behaviors among 6–11 Year Old Schoolchildren in Kosovo. Acta Stomatol. Croat..

[45] Chen L., Hong J., Xiong D., Zhang L., Li Y., Huang S., Hua F. (2020). Are parents’ education levels associated with either their oral health knowledge or their children’s oral health behaviors? A survey of 8446 families in Wuhan. BMC Oral Health.

[46] Pargaputri A., Maharani A., Patrika F. (2023). Dental and oral health education in parents of Taam Avicenna playgroup students. J. Pemberdaya. Publ. Has. Pengabdi. Kpd. Masy..

[47] Buldur B., Oguz E. (2023). Reliability and validity of the Turkish version of the Oral Health Literacy Assessment Task: Pathways between parental oral health literacy and oral health consequences in children. Int. J. Paediatr. Dent..

[48] Litchford A., Savoie Roskos M.R., Wengreen H. (2020). Influence of fathers on the feeding practices and behaviors of children: A systematic review. Appetite.

[49] Babaei A., Pakdaman A., Hessari H., Shamshiri A.R. (2019). Oral health of 6–7 year-old children according to the Caries Assessment Spectrum and Treatment (CAST) index. BMC Oral Health.

[50] Lambert M.J., De Visschere L.M.J., Martens L.C., Deschepper E., Vanobbergen J. (2019). The impact of a prospective 4-year longitudinal school intervention for improving oral health and oral health inequalities in primary schoolchildren in Flanders-Belgium. Int. J. Paediatr. Dent..

[51] Aslan Ceylan J., Aslan Y., Ozcelik A.O. (2022). The effects of socioeconomic status, oral and dental health practices, and nutritional status on dental health in 12-year-old school children. Egypt. Pediatr. Assoc. Gaz..

[52] Bae J.H., Obounou B.W.O. (2018). Presence of Dental Caries Is Associated with Food Insecurity and Frequency of Breakfast Consumption in Korean Children and Adolescents. Prev. Nutr. Food Sci..

[53] Hill B. (2020). Evaluating the association between food insecurity and dental caries in US children 1–19 years: Results from the National Health and Nutrition Examination Survey (NHANES) 2013-2014. J. Public Health Dent..

[54] Al-Qahtani S.M., Razak P.A., Khan S.D. (2020). Knowledge and Practice of Preventive Measures for Oral Health Care among Male Intermediate Schoolchildren in Abha, Saudi Arabia. Int. J. Environ. Res. Public Health.

[55] Angelopoulou M., Kavvadia K., Oulis C., Reppa C. (2015). Oral Hygiene Facilitators and Barriers in Greek 10 Years Old Schoolchildren. Int. J. Clin. Pediatr. Dent..

[56] Nagashima Y., Shigeishi H., Fukada E., Amano H., Urade M., Sugiyama M. (2018). Self-check with plaque disclosing solution improves oral hygiene in schoolchildren living in a children’s home. Arch. Public Health.

[57] Mahesh Kumar P., Joseph T., Varma R.B., Jayanthi M. (2005). Oral health status of 5 years and 12 years school going children in Chennai city—An epidemiological study. J. Indian. Soc. Pedod. Prev. Dent..

[58] Quadri M.F., Shubayr M.A., Hattan A.H., Wafi S.A., Jafer A.H. (2018). Oral hygiene practices among Saudi Arabian children and its relation to their dental caries status. Int. J. Dent..

[59] Sălcudean A., Cîmpian D.-M., Popovici R.-A., Forna N., Corodan-Comiati D.-M., Sasu A.-B., Cozma M.-M., Bodo C.-R., Enache E.-C., Păcurar M. (2025). Dietary Habits and Their Influence on the Microbiome and Mental Health in Adolescents. Nutrients.

[60] Sălcudean A., Bodo C.-R., Popovici R.-A., Cozma M.-M., Păcurar M., Crăciun R.-E., Crisan A.-I., Enatescu V.-R., Marinescu I., Cimpian D.-M. (2025). Neuroinflammation—A Crucial Factor in the Pathophysiology of Depression—A Comprehensive Review. Biomolecules.

[61] Halonen H., Pesonen P., Seppä L., Peltonen E., Tjäderhane L., Anttonen V. (2013). Outcome of a Community-Based Oral Health Promotion Project on Primary Schoolchildren’s Oral Hygiene Habits. Int. J. Dent..

[62] Jordão L.M., Vasconcelos D.N., Moreira Rda S., Freire Mdo C. (2012). Individual and contextual determinants of periodontal health in 12-year-old schoolchildren in a Brazilian capital city. Int. J. Dent..

[63] Ősz B.E., Jîtcă G., Sălcudean A., Rusz C.M., Vari C.E. (2023). Benzydamine-An Affordable Over-the-Counter Drug with Psychoactive Properties-From Chemical Structure to Possible Pharmacological Properties. Pharm..

[64] Suwarsono S., Safitri L., Sunarjo L. (2023). Dental health educational: Media videos and animated videos on increasing dental and oral health maintenance behavior. JCHAT.

[65] Puteri A., Emini E. (2023). Storytelling as increasing knowledge of dental health in kindergarten. JCHAT.

[66] Sălcudean A., Popovici R.A., Pitic D.E., Sârbu D., Boroghina A., Jomaa M., Salehi M.A., Kher A.A.M., Lica M.M., Bodo C.R. (2025). Unraveling the Complex Interplay Between Neuroinflammation and Depression: A Comprehensive Review. Int. J. Mol. Sci..

[67] Sălcudean A., Osz B.E., Bodo C.R., Muntean D.L. (2024). Serum serotonin level can be used as a predictive marker for depression in patients with type 2 diabetes mellitus. True or false?. Farmacia.

[68] Feier A.M., Portan D., Manu D.R., Kostopoulos V., Kotrotsos A., Strnad G., Dobreanu M., Salcudean A., Bataga T. (2022). Primary MSCs for Personalized Medicine: Ethical Challenges, Isolation and Biocompatibility Evaluation of 3D Electrospun and Printed Scaffolds. Biomedicines.

[69] Zhao S., Wu Y. (2024). Knowledge, attitudes and practices among patients with periodontal disease toward disease management. Front. Public Health.

[70] Asa’ad F.A., Rahman G., Al Mahmoud N., Al Shamasi E., Al Khuwaileidi A. (2015). Periodontal disease awareness among pregnant women in the central and eastern regions of Saudi Arabia. J. Investig. Clin. Dent..

[71] Sălcudean A., Cîmpian D.M., Popovici R.A., Vasiesiu A.M., Muntean D.L. (2025). Pharmacological insights with impact on depressive symptoms. Farmacia.

[72] Krauss B.A., Leroy P.L., Krauss B.S. (2024). Establishing trust with children. Eur. J. Pediatr..

[73] Sălcudean A., Olariu I., Cincu M.-G., Popovici R.A., Comșulea I., Bodo C.-R., Cîmpian D.-M., Strete E.-G. (2025). The Association Between Borderline Personality Disorder Symptoms and Social Behaviour Among University Students. Medicina.

[74] Sălcudean A., Truşculescu L.M., Popovici R.A., Şerb N., Paşca C., Bodo C.R., Crăciun R.E., Olariu I. (2024). Dental anxiety—A psychosocial cause affecting the quality of life—a systematic review. Rom. J. Oral Rehabil..

[75] Bleyen S., Mooens K., Jancic L., Lambrechts P., Meschi N., Pedano M.S. (2025). Virtual reality for patients with dental anxiety: A scoping review. J. Dent..

[76] Strete E.-G., Cincu M.-G., Sălcudean A. (2025). Disordered Eating Behaviors, Perceived Stress and Insomnia During Academic Exams: A Study Among University Students. Medicina.

[77] Kettle J., Porritt J., Baker S.R., Rodd H., Cross E., Marshman Z. (2025). Your Teeth, You Are in Control: A Process Evaluation of the Implementation of a Cognitive Behavioural Therapy Intervention for Reducing Child Dental Anxiety. Community Dent. Oral Epidemiol..

[78] Ilea M., Forray A.-I., Petrescu N.B., Mirica I.-C., Ormenişan A., Üçtaşli M.B., Melnic A., Lucaciu O.P. (2025). COVID-19 and Its Aftermath on Pediatric Oral Health: A Study of Dental Caries and Hygiene in Romanian Children. Children.

[79] Funieru C., Nicolescu M.I., Băicuş C., Slușanschi O., Bica C.I., Moldoveanu A., Perpelea A.C., Țandără A.T. (2024). Oral Hygiene Profile of Schoolchildren from Bucharest, Romania—How It Can Be Used and Improved for Better Prevention of Oral Diseases. Healthcare.

[80] Sfeatcu R., Cărămidă M., Sava-Rosianu R., Matichescu L.M., Galuscan A., Dumitrache M.A. (2023). Carious status and socio-behavioral risk factors among 12 year-old children in South-Central region in Romania. BMC Oral Health.

[81] Perpelea A.-C., Sfeatcu R., Tănase M., Meleșcanu Imre M., Ripszky Totan A., Cernega A., Funieru C., Pițuru S.-M. (2024). A STEPwise Approach for Oral Hygiene Behavior of Schoolchildren in Romania. Healthcare.

[82] Yang F., SuoNan L., Gong M. (2025). A cross-sectional investigation on oral health and hygiene among children aged 7–10 years in extremely high altitude areas of China. Sci. Rep..

[83] Bayraktar Nahir C., Coşgun A., Şahin N.D., Altan H. (2025). Primary school children’s oral hygiene knowledge assessed with different educational methods: A cross-sectional study. BMC Oral Health.

[84] Buunk-Werkhoven Y.A.B., Tamulienė R., Mačiulienė D. (2025). Exploring parental opinions on oral hygiene behavior and knowledge of their young children in Lithuania: A cross-sectional survey study. Front. Oral Health.

[85] George A., Sousa M.S., Kong A.C., Blinkhorn A., Patterson Norrie T., Foster J., Dahlen H.G., Ajwani S., Johnson M. (2019). Effectiveness of preventive dental programs offered to mothers by non-dental professionals to control early childhood dental caries: A review. BMC Oral Health.

